# Development of quality indicators to measure pre-hospital emergency medical services for road traffic injury

**DOI:** 10.1186/s12913-021-06238-1

**Published:** 2021-03-16

**Authors:** Saber Azami-Aghdash, Ahmad Moosavi, Hojatolah Gharaee, Ghader Sadeghi, Haleh Mousavi Isfahani, Alireza Ghasemi Dastgerdi, Mohammad Mohseni

**Affiliations:** 1 Road Traffic Injury Research Center, Tabriz University of Medical Sciences, Tabriz, Iran; 2 Tabriz Health Services Management Research Center, Health Management and Safety Promotion Research Institute, Tabriz University of Medical Sciences, Tabriz, Iran; 3 Department of Health and Community Medicine, Dezful University of Medical Sciences, Dezful, Iran; 4 District Health Center of Hamadan City, Hamadan University of Medical Sciences, Hamadan, Iran; 5 School of Health Management and Information Sciences, Iran University of Medical Sciences, Tehran, Iran; 6 Disaster and Emergency Medical Management Center, Isfahan University of Medical Sciences, Isfahan, Iran; 7 Health Management and Economics Research Center, Iran University of Medical Sciences, Tehran, Iran

**Keywords:** Quality measurement indicators, Pre-hospital, Emergency care, Road traffic injuries

## Abstract

**Background:**

Pre-Hospital Emergency Care (PEC) is a fundamental property of prevention of Road Traffic Injuries (RTIs). Thus, this sector requires a system for evaluation and performance improvement. This study aimed to develop quality indicators to measure PEC for RTIs.

**Methods:**

Following the related literature review, 14 experts were interviewed through semi-structured interviews to identify Quality Measurement Indicators (QMIs). The extracted indicators were then categorized into three domains: structure, performance, and management. Finally, the identified QMIs were confirmed through two rounds of the Delphi technique.

**Results:**

Using literature review 11 structural, 13 performance, and four managerial indicators (A total of 28 indicators) were identified. Also, four structural, four performance, and three managerial indicators (A total of 11indicators) were extracted from interviews with experts. Two indicators were excluded after two rounds of Delphi’s technics. Finally, 14 structural, 16 performance and, seven managerial indicators (A total of 37indicators) were finalized.

**Conclusion:**

Due to the importance and high proportion of RTIs compared to other types of injuries, this study set out to design and evaluate the QMIs of PEC delivered for RTIs. The findings of this research contribute to measuring and planning aimed at improving the performance of PEC.

**Supplementary Information:**

The online version contains supplementary material available at 10.1186/s12913-021-06238-1.

## Background

Road Traffic Injuries (RTIs) is one of the major public health concerns worldwide [1–3]. It is estimated that each year 1.35 million people die from RTIs worldwide, and more than 50 million people get injured [4]. According to the results of the Global Burden of Disease Study, RTIs is the 8th leading cause of death, and cause about 2.46% of all deaths worldwide [5]. Also, based on monitoring reports of Millennium Development Goals (MDGs), RTIs account for around a quarter (24%) of all injury-related deaths [6].

One of the main strategies to reduce the burden of injuries, especially caused by RTIs, is investing on and developing Prehospital Emergency Care (PEC) [7–9]. PEC ranged from a patient’s bedside in the community to a hospital emergency [10]. Quick, efficient, and effective PEC can save the lives of many patients at vital moments [11]. Finding high-risk patients as soon as possible and providing appropriate treatment is one of the main goals of PEC [12].

In many countries around the world, especially in Low- and Middle-Income Countries (LMICs), PEC has less developed, and health system performance in this area is not satisfactory [13, 14]. In Iran, despite significant progress in PEC, such as increasing the number of ambulance dispatch sites and the number of ambulances, providing better and high-quality equipment, increasing in the number of staff, developing better educational plans for PEC team members, and adding helicopter and motorcycle ambulances to the EMS, there are many problems and shortcomings [15, 16].

Therefore, like other sectors of the health system, PEC requires a monitoring and evaluation mechanism in order to improve the performance and quality of care [17, 18]. Different models and methods may be used to evaluate the performance of PEC [19, 20]. Quality Measurement Indicators (QMIs) is one of the most important methods [21–23]. The QMIs usually provide quantitative outputs that could be used as a standard or guideline for improvement of service quality [24, 25]. In recent years, several attempts have been made to develop QMIs for PEC, especially time intervals indicators [26–28]. Howard et al., (2019), through a 3-round modified Delphi technic, identified 90 clinical Quality Indicators (QIs) in 15 subcategories, and 14 non-clinical QIs in two subcategories for PEC in South Africa [24]. Through a scoping review study, Howard et al. (2018) investigated the characteristics and development methods of the QIs in the field of PEC, who identified 331 QIs by the article review and 15 by the website review [29]. Similarly, there are several published studies [25, 30–33] that develop indicators for measuring the performance of PEC.

To date, few comprehensive and specific studies have been published about the development of indicators for RTIs. Given the high prevalence of traffic accidents and injuries, and considering the important role of the PEC in reducing the complications and burden of RTIs, specific indicators to measure the performance of PEC can have a significant impact on improving the quality and effectiveness of these cares. Therefore, this study set out to develop the QMIs of PEC delivered to RTIs in Iran.

### PEC in Iran

Table 1 provides an overview of the structural characteristics of PEC in Iran.

**Table 1:** Structural Characteristic of Prehospital Emergency Care in Iran

Population coverage (Iran’s population)^a^ (based on the 2017 census)	Total population (79926270), Sex (Male 50.7%, Female 49.3%), Residence (Urban 79.4%, Rural 25%, other 0.6%)
PEC^b^ history	In 1975, as the fourth country worldwide, Iran launched PEC with seven sites.
EMS^c^ Provider Organization	Dependent on Disaster and Emergency Medical Management Centers (DEMMCs), National Emergency Organization, and Ministry of Health
EMS Call Number	115, free call
EMS have Independent CC^d^	Yes
Composition of EMS Dispatch Center’s staff and Qualification	1-Paramedic Call Taker (having BSc in EMS) 2- Paramedic (having BSc in EMS/ Nursing) 3- Supervisor (M.D.)
Type of Ambulance Crews and Qualification	1- Paramedic (having BSc in EMS/ Nursing) 2- Driver (license in BLS as the first responder)
TATV^e^	Ambulance Buses: 5500 Ambulance Motorcycles: 500 Ambulance Helicopters: 42 Ambulance Emergency relief boats:2
Emergency Stations	2190
Number of missions per year	3.8 to 4 million
Process	1. **Call 115 for help:** This number is the same throughout the country and rings in the control room of the emergency station 2. **Triaged in CC:** The command room staff includes a physician, nurse, and wireless operator, in which nursing experts respond to calls. The task of these experts is to take a telephone history of the patient’s condition, and if the patient’s history shows a state of emergency, they will try to get the location of the accident, then send the rescue unit. 3. **Send the nearest rescue unit to the scene:** The emergency command room calls the nearest rescue unit to the location of accident, to do the mission. The geographical area covered by each center is defined by distances (in rural and urban roads) and population (in cities). 4. **Reach the relief unit to the location of accident and take immediate actions in the scene** 5. **Putting the patient in the ambulance and move toward the medical center:** Immediately after this step, the patient’s history is communicated by the doctor of the command room, using a telephone or wireless machine, and the doctor guides the personnel regarding the required care and medication. 6. **Delivery of the patient to the medical center:** When the rescue unit arrives at the medical center, the patient is handed over to the doctor or emergency manager by giving history and filling out the mission report form, and this is where the rescue unit’s mission ends.

^a^According to Iranian law, the PEC must cover all the Iran’s population, ^b^*PEC* Prehospital Emergency Care, ^c^*EMS* Emergency Medical Services, ^d^*CC* Call Center, ^e^*TATV* Type of Ambulance Transportation Vehicle

## Method

This is a qualitative study conducted using Grounded Theory (GT) approach in 2020 in Iran. The strength of this approach is that it’s an inductive research method with a qualitative approach that is particularly useful to gain insight into topics that have not been comprehensively studied before, and our knowledge about it, is limited [34, 35].

This study was conducted in three steps, each of which is described separately.

### Step one: to extract the QMIs of PEC delivered to RTIs using a literature review

At this step, required data were collected using search of the keywords including “road traffic accident”, “Road traffic accidents”, “road accident”, “motorcycle accident”, “motorcycle accident”, “motorcycle accident”, “motor vehicle accident”, “motor vehicle accident”, “road traffic collision”, “indicator”, “index”, “pre-hospital”, “Emergency Medical Services” in different databases. The English studies were searched in PubMed, Scopus, Google scholar; and the Persian studies in SID and MagIran (the Persian databases) (search strategies in PubMed and Scopus are presented in Additional file 1). The other available information sources were also searched using Manual search of selected journals, reference checks (reference of reference), review of organizational reports, published government documents, websites, etc. from January 1990 to June 2020. Only studies and documents that referred to QMIs in PEC were included in the study. Studies that had not reported topics or other information related to QMIs in PEC, studies which were not related to PEC (for example the studies that report the indicators of the emergency department or clinical indicators) or RTIs (like studies focusing on other types of prehospital care, such as heart attacks and other types of trauma) were excluded. The data were analyzed manually and summarized using the Extraction Table.

### Step two: to extract the QMIs of PEC delivered to RTIs using semi-structured interview with experts

The setting of this phase of the study was the Iranian Road Emergency Organization, Ministry of Roads & Urban Development, Ministry of Health, Medical universities, Iranian Traffic Police (Agency), Iranian Legal Medicine Organization, Iranian Red Crescent Society, and related research centers. Semi-structured interviews were conducted with participation of officers, technicians and employees of Disaster and Emergency Medical Management Centers (DEMMCs) and faculty members who had extensive research and knowledge in the field of RTIs.

Criteria for selecting the subjects were as follows:

Having published books, papers or other research works in the field of PEC and RTIs for faculty members and head of research centers, having at least 2 years of work experience in the field of DEMMCs, having Iranian nationality, being fluent in speaking Persian language, having at least high school degree, and having the desire and ability to participate in the study.

Purpose-based sampling was used to select participants, in which individuals who have the most and richest information, and able to provide information properly, will select as the participants [36–38]. Sampling was continued until information saturation, the point in which the researchers felt that new information would not be obtained as sampling continued. This level was achieved in the present study with 14 participants. To get the diverse data, it was decided that the selected participants have variety regarding age, employment status, work experience, educational qualification, and job position.

Semi-structured interviews were carried out in Persian. Interviews were conducted in a comfortable place for the participants. The interviews were carried out using the guide questions (Additional file 2), which were designed through the literature review and expert opinions. Interviews lasted 45 to 90 min. Interviews were recorded on a digital audio recorder, and the researcher also took notes to record information. The recorded file immediately after each interview was listened to several times and transcribed by researchers.

The data was analyzed using Conventional Content-Analysis (CCA), which is a method for identifying, analyzing, and reporting patterns within the text and is widely used in qualitative data analysis [39–41]. In most studies, Donabedian’s model for quality of care [42, 43], have been used to categorize the indicators, which includes the structure, process, and outcome areas. However, in the present study, based on the results of literature review, expert opinions, and opinions of research team members, indicators were categorized into three areas of structural indicators (equivalent to structure indicator of Donabedian’s model), performance indicators (equivalent to process and outcome indicators of Donabedian’s model) and management indicators.

Peer checks, expert checks, immersed (deep involvement of the researcher/s with the aim and the process of study) and response validity (at the end of each interview, the participants’ statements were summarized and restated to confirm the researchers’ notes and perception by interviewee) were used for rigor, and data transferability and reliability.

Oral informed consent was obtained from participants, and participants were allowed to withdraw from the study at any time. Also, the objectives of the study were explained to the participants, at first.

### Step three: confirmation of the QMIs of PEC delivered to RTIs via Delphi technique

**Fig. 1:**
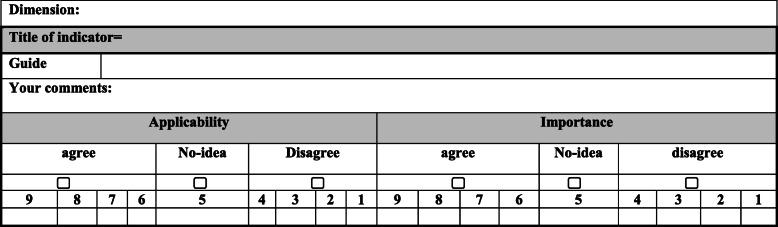
Delphi Form

The validity of the QMIs of PEC delivered to RTIs was confirmed using the Delphi technique. The modified Delphi form that had been applied in the previous study of researchers was used in this study [44] (Fig. 1). This form includes three dimensions of structural, performance, and managerial, the title of indicators, description help composed of a brief explanation about the indicators and how to measure it, experts’ comments about the indicators, and scoring section. Each expert scores indicators from two aspects of importance (Is this indicator important and should be taken into consideration?) and applicability (How much is it possible to collect information for this indicator?). In this section, the experts firstly expressed their general opinion by choosing one of the three “disagree”, “no idea” and “agree” options. Then, based on their previous choice, they scored each indicator from 1 to 9 (1 to 4 disagree, 5 no idea, and 6 to 9 agree). The indicators that scored as 7 or higher were accepted. Indicators with a mean score of 4 to 7 went to the second round of Delphi and indicators with a mean score of less than 4 were excluded. The Delphi questionnaire consisted of three parts: a brief introduction about the aims and necessities of the study, a description guide for completing the form, and a scoring section. Delphi forms were sent to the experts via email. They were given 2 weeks to complete the form. After 2 weeks a reminder email was sent again.

## Results

Table 2 presents the characteristics of the participants. From the table it can be seen that all the participants were male, the mean age of participants was 41 years, the mean work experience was about 11 years, and 5 participants had work experience in a rural setting.

**Table 2:** The characteristics of the participants (*N* = 14)

Participant number	Sex	Age	work experience	Job	Work experience in a rural setting	Participant number	Sex	Age	work experience	Job	Work experience in a rural setting
1	Male	43	18	Emergency medical technician	Yes	8	Male	50	22	Faculty member	NO
2	Male	33	8	Emergency medical technician	NO	9	Male	45	10	Faculty member	NO
3	Male	49	19	Emergency medical technician	Yes	10	Male	31	2	Faculty member	NO
4	Male	29	2	Emergency medical technician	Yes	11	Male	38	4	Faculty member	NO
5	Male	36	5	Emergency medical technician	NO	12	Male	48	12	Faculty member	NO
6	Male	45	15	Disaster and Emergency Medical Management Centers (DEMMCs) officers	NO	13	Male	45	13	DEMMCs employee	NO
7	Male	52	25	DEMMCs officers	Yes	14	Male	29	3	DEMMCs employee	Yes

### Results of the literature review

**Fig. 2:**
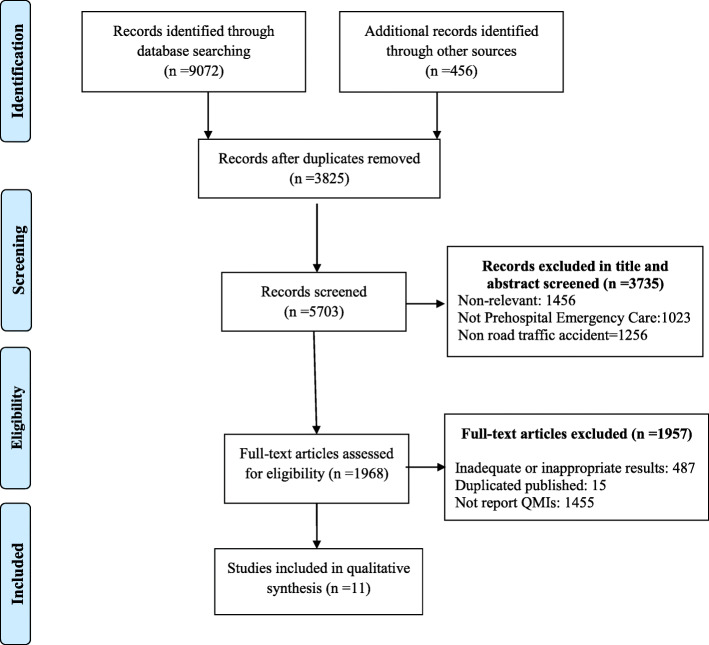
The screening process and selection of articles

Out of 9128 documents that were found through search in databases and other sources of information, 3825 cases were excluded due to duplication between databases. Through the screening of the title and abstract, 3735 documents were excluded. In the full-text eligibility review, 1957 cases were excluded, and finally, 11 documents were included (Fig. 2) (Additional file 3 provides the list of the included documents) [8, 24, 28, 45–52].

Out of the 11 documents included, nine were articles and two were reports. The studies were mostly conducted in the USA (four studies). Finally, 207 indicators were extracted. After removing the duplicates, merging similar items, and analyzing the indicators by the research team’s members, 28 indicators were finalized. The indicators extracted through the literature review are summarized in Table 3. 11 indicators were categorized as structural indicators, 13 as the performance indicators, and 4 as the management indicators.

**Table 3:** List of indicators extracted using literature review to measure the quality of pre-hospital Emergency Medical Services (EMS) provided to Road Traffic Injuries (RTIs)

Dimension	Title of indicators
**Structural indicators**	1. Road Emergency Stations coverage rate
	2. Urban Emergency Stations coverage rate
	3. Motorcycle ambulance coverage rate
	4. Ambulance bus coverage rate
	5. Number of backup ambulances
	6. The ratio of active ambulances to the total number of ambulances
	7. Active manpower
	8. Status of pre-hospital emergency stations’ building
	9. Independence of pre-hospital emergency stations building
	10. The ownership of the pre-hospital emergency stations building
	11. Number of standard pre-hospital emergency stations in terms of building size
**Performance indicators**	1. The average time between an emergency call up to notification to operations dispatch and guidance unit
	2. The average time between receiving a mission by 800 to notify the stations
	3. The average time between the notification of mission to stations up to the ambulance dispatch
	4. Average Scene Time (the time is spent on the accident scene)
	5. The average time between the moment the ambulance move from the accident scene up to arrive at the hospital in urban missions (Transport time)
	6. The average time between the moment the ambulance move from the accident scene up to the arrive at the hospital in road missions (Transport time)
	7. Duration of ambulance stop in hospital (Hospital time)
	8. Per capita missions performed for each active urban ambulance
	9. Coverage rate of the rural emergency stations
	10. Helicopter coverage rate
	11. Patient satisfaction
	12. Successful CPR rate
	13. Death rate during transfer
**Management indicators**	1. Management visits from pre-hospital emergency stations
	2. Training courses in the field of trauma care
	3. The average distance between pre-hospital emergency centers (kilometers)
	4. Average Distance between Pre-hospital Emergency Centers to Red Crescent Emergency Centers (kilometers)

### Results of the interview with experts

The indicators extracted from the interview with experts, are set out in Table 4. At this phase, four indicators in the field of structure, four indicators in the field of performance, and three indicators in the field of management (11 indicators in total) were added (The number of indicators extracted in the interviews were more than those listed in Table 4, but due to overlap with the indicators found using the literature review, were not presented in this section).

**Table 4:** Indicators extracted from interviews to measure the quality of pre-hospital Emergency Medical Services (EMS) provided to Road Traffic Injuries (RTIs)

Dimension	Title of indicators
**Structural indicators**	1. Rural emergency stations coverage rate
	2. The ratio of manpower who have an academic degree in emergency medicine
	3. Number of standard pre-hospital emergency stations in terms of safety
	4. Number of pre-hospital emergency stations that have standard ambulance nest
**Performance indicators**	1. Number of consultations by the nurse
	2. The number of consultations referred to the physician
	3. The dispute between pre-hospital emergency personnel and hospital emergency personnel
	4. Number of cases of change/return from the hospital
**Management indicators**	1. Evaluation of stations’ personnel performance
	2. Assessment of staff satisfaction
	3. The ratio of the personnel number to the missions number

### Indicators confirmation results

According to the results of the first round of Delphi, out of 39 indicators, two indicators were excluded (rural stations coverage rate from structural dimension, and percentage of rural emergency stations coverage from performance dimension), two indicators entered the second round of Delphi and 35 indicators were accepted. Based on the scores of two indicators in the second round of Delphi, the indicators were included. So Delphi finished in the second round. The list of finalized indicators is shown in Table 5.

**Table 5:** Final List of indicators to measure the quality of pre-hospital Emergency Medical Services (EMS) provided to Road Traffic Injuries (RTIs)

Dimension	Title of indicators	Numerators of indicators	Denominators of indicators	Description
**Structural indicators**	1. Urban emergency stations coverage rate	Number of urban stations	Number of required urban stations based on standards	One station for every 40 km with an ambulance
	2. Urban Emergency Stations Coverage rate	Number of available urban stations	Number of required urban emergency stations based on standards	–
	3. Motorcycle ambulance coverage rate	Number of available motorcycle ambulance	Number of required motorcycle ambulance based on standards	In cities with a population of over 250,000 and one motorcycle ambulance unit for every 4 urban stations
	4. Ambulance bus coverage rate	Number of available ambulance bus	Number of required ambulance bus based on standards	An ambulance bus for one million people covered
	5. Number of backup ambulances	Number of available backup ambulances	Number of active stations	One backup ambulance for every 3 stations
	6. The ratio of active ambulances to the total number of available ambulances	Number of active ambulances	Number of ambulances delivered to that center	An active ambulance means an ambulance that is currently serving in pre-hospital emergency stations. (Support ambulances, under maintenance, headquarters’ vehicles are not considered for this indicator.)
	7. Active manpower	Number of employed manpower per station	Required Manpower for each station	Standard Number of manpower per station = 9.25
	8. Pre-hospital emergency building’ status	Number of pre-hospital emergency stations located in a building	Total number of pre-hospital emergency stations	The purpose of this indicator is to determine the building status, based on using the prefabricated building or Conex as a station.
	9. Independence of pre-hospital emergency stations’ building	The number of pre-hospital emergency stations whose building (of any kind) is completely independent.	The total number of pre-hospital emergency stations.	Independence means that the station’s building is not part of a hospital, fire department, etc.
	10. The ownership status of the pre-hospital emergency stations’ building	Number of pre-hospital emergency building owned by the country’s emergency services	Total number of pre-hospital emergency stations.	The purpose of this indicator is to measure the ratio of stations’ building owned by country’s emergency services to the stations which are rented by the country’s emergency services
	11. Number of standard pre-hospital emergency stations in terms of building size	The number of standard pre-hospital emergency stations in terms of building size.	The total number of pre-hospital emergency stations.	Road station standard: at least 87 m
	12. The ratio of manpower having an academic degree in emergency medicine	The number of manpower with an academic degree in emergency medicine in active pre-hospital emergency stations.	The total number of manpower employed in active pre-hospital emergency stations.	The purpose of this indicator is to measure the status of using manpower other than who have emergency medicine degree (nursing, anesthesia, etc.)
	13. Number of standard pre-hospital emergency stations in terms of safety	The number of pre-hospital emergency stations that have at least one safety system/tool.	Total number of pre-hospital emergency stations	–
	14. Number of pre-hospital emergency stations that have standard ambulance site	The number of pre-hospital emergency stations with ambulance nest.	Total number of pre-hospital emergency stations	–
**Performance indicators**	1. The average time between the emergency call of 115 up to notification to 800	Total time of emergency missions from call emergency 115 to notification to 800.	Number of missions announced	–
	2. The average time between receiving a mission by 800 to notify the stations	Total time between receiving the mission by 800 to notification of the mission to the emergency station	Number of missions announced	–
	3. The average time between the mission announcement to stations until the departure of the ambulance	Total time between the mission announcement to the station until the ambulance leaves the station	Number of missions announced	–
	4. The average time spent in the accident scene (Scene Time)	Total time spent at the scene of the accident	Number of missions announced	Scene Time: the time between reaching the scene of the accident and the time of leaving the scene of the accident toward the hospital
	5. The average time between the ambulance departures from the scene of the accident to the hospital until the moment of arrival at the hospital on urban missions (Transport time)	Total time of emergency missions, between ambulance departures from the accident scene to arrival at the target hospital in urban missions.	Number of city missions leading to departure to the hospital	–
	6. The average time between the ambulance departure from the scene of the accident to the hospital until the arrival at the hospital in the road missions (Transport time)	Total time of emergency missions between the departures from the scene of the accident to arrival at the target hospital in the road missions.	Number of road missions leading to hospitalization	–
	7. Duration ambulance stop in hospital (Hospital time)	Total time of ambulance stops in the hospital	Number of missions leading to departure to the hospital	–
	8. Per capita missions did for each active urban ambulance	The total number of urban missions in the specified coverage area.	Total number of active urban ambulances	–
	9. Helicopter cover rate	The total number of missions performed by the helicopter.	The total number of air missions done in the covered area.	An area of two kilometers around the helicopter site in the emergency station
	10. Patient satisfaction	The number of service recipients who are satisfied with pre-hospital emergency services.	All recipients of pre-hospital emergency services	–
	11. Successful CPR rate	Number of successful CPR recorded in prehospital emergency	Total number of patients transferred by a pre-hospital emergency during cardiopulmonary resuscitation	–
	12. The death rate during transfer	The number of deaths in traffic accidents during transfer to hospital by pre-hospital emergency	The total number of injured in traffic accidents who transferred by pre-hospital emergency.	All traffic injured died in the ambulance during transfer to the hospital
	13. Number of consultations by the nurse	Total number of calls that have been given consultation by the nurse	Total number of calls with the pre-hospital emergency	–
	14. The number of consultations transferred to the physician	Total number of calls that have been given consultation by the physician	Total number of calls with the pre-hospital emergency	–
	15. The dispute between pre-hospital emergency personnel and hospital emergency personnel	Total Number of disputes between pre-hospital emergency and hospital emergency personnel.	The total number of missions leading to departure to the hospital.	The dispute refers to the cases in which that reception unit has been forced to intervene.
	16. Number of return/hospital change	The total number of cases that have led to the change of hospital due to improper hospital selection.	The total number of missions leading to departure to the hospital.	Improper hospital: means that for any reason, such as the lack of empty beds, specialists, necessary facilities, etc., pre-hospital emergency technicians are forced to change hospitals.
**Management indicators**	1. Management visits from pre-hospital emergency stations	The total number of pre-hospital emergency stations that is visited by management at least once a year (by a checklist).	Total number of pre-hospital emergency stations	–
	2. Training courses in the field of trauma care	Total number of personnel who have passed at least one training course in trauma care in one past year	Total number of personnel working in pre-hospital emergency stations	–
	3. The average distance between pre-hospital emergency stations (kilometers)	–	–	–
	4. Average Distance of Pre-hospital Emergency to nearest Red Crescent Emergency Center (kilometers)	–	–	–
	5. Evaluate the performance of stations’ personnel	The total number of personnel whose performance has been evaluated at least once in one past year using a checklist.	Total number of personnel working in pre-hospital emergency stations	–
	6. Personnel satisfaction assessment	Total number of personnel whose satisfaction rate has been assessed at least once in one past year	Total number of personnel working in pre-hospital emergency stations	–
	7. number of personnel to the number of missions ratio	The total number of personnel working at each station.	The average number of missions performed by each station	–

## Discussion

In this study, using literature review, interview with experts and Delphi technique, 37 QMIs (14 structural indicators, 16 performance indicators, and 7 management indicators) of PEC delivered to RTIs were developed.

Since a high percentage of pre-hospital emergency missions are because of RTIs, developing, and measuring specific indicators in this area can be very helpful. Regarding this, in this study, to evaluate the performance of PEC, QMIs was developed exclusively in the field of RTIs. However, according to the literature review, there has been little discussion about this topic, and most studies introduced general indicators for evaluating the performance of PEC. For example, Iran’s emergency organization has introduced 16 indicators at the national level and 13 indicators at the university level, to evaluate the performance of PEC [53]. Various studies conducted in other countries are limited to developing general indicators, and specific indicators in the field of traffic accidents were less considered [25, 33, 54]. It should be noted that the aim of this study was not to develop a separate set and analysis system for RTIs indicators. Rather, we aimed to put more emphasis on RTIs indicators. It is expected that if there are specific indicators for PEC in RTIs, planning, and implementing interventions to improve the quality of services will be more effective.

In the present study, we used three phases of literature review, experts’ opinion review, and the Delphi technique. These three steps are one of the most common procedures for developing indicators [22, 55, 56].

An important set of PEC QMIs, which have been reported in many studies, are the indicators for measuring the time between the emergency call and arrival at the scene of accidents [57–59]. Timely arrival at the patient’s bedside is a very important factor to increase the chance of patients’ survival, and reduce the side effects of accident [60, 61]. This finding broadly supports the results of other studies in this area which confirm that ambulance’s arrival under five minutes for RTIs and less than eight minutes for heart patients will cause a significant reduction in mortality and other complications [62–64]. However, the results of many studies have shown that response time in many countries, especially in LMICs, is higher than the international standards [65–68]. This problem can be somewhat eliminated by familiarizing pre-hospital emergency technicians with suburban and urban routes, using satellite and GIS systems, educating the community to open the path for ambulances, constructing dedicated routes, and using Motorcycles. The number of developed indicators in the field of RTIs (six indicators in the performance dimension) could be attributed to the great importance of these indicators in this area.

There are two reasons for paying special attention to management indicators, in the present study. The first reason may be that the proper management has a considerable impact on pre-hospital emergency performance. Little published research on management indicators is the second reason. This also accords with our earlier mini-review study (2017), about the performance of pre-hospital emergency indicators in Iran and the causes of delay in arrival at the scene of the accident, which showed that many causes of delay can be solved by proper management [69]. Jarrel and colleagues (2007) also showed that one of the main reasons for the increase in pre-hospital emergency performance times is poor management in the distribution of ambulances and stations [70]. In addition to the importance and impact of proper management on the quality and performance of pre-hospital emergency services, improving management compared to other interventions, such as providing ambulances or other equipment, or increasing manpower, will costs less and will affect in very shorter time. It can thus be suggested that special attention should be paid to proper management in this area. Also, there is abundant room for further studies on the development of pre-hospital emergency management indicators.

Although based on the results of the literature review and our best knowledge, this is the first study that has developed specific and comprehensive QMIs for PEC performance in RTIs, the findings in this study are subject to at least two limitations. First, the most important limitation lies in the fact that participants of the present study were limited to Iranian experts and stakeholders. This issue can make the generalizability and usability of indicators in other countries difficult. Another limitation of the present study was that due to the outbreak of the Coronavirus disease (COVID-19), we were unable to validate the developed indicators through validity analysis after their utilization. To overcome these limitations, it is recommended that managers and policymakers localize the indicators following the local conditions of their own country and check it’s validity before deciding to use these indicators. It is also suggested that in the future similar studies, researchers consider the conditions of other countries and use the international stakeholders and experts’ views. Also, a comprehensive online registry system can be effective in validating the indicators (which unfortunately did not happen in the present study).

## Conclusion

As a result of this study, 37 quality indicators, to measure the pre-hospital emergency medical services for RTIs, were developed in dimensions of the structure, performance, and management. Due to the importance and high proportion of RTIs compared to other types of injuries, this study suggests that more attention should be paid to these indicators. It is recommended that managers and policymakers use these indicators as a tool to measure and improve the performance of pre-hospital emergencies, after localizing and validating them based on local conditions.

## Supplementary Information

**Additional file 1.** Search strategy up to June 13st, 2020.

**Additional file 2.** Interview Guide.

**Additional file 3.** List of review document.

## Data Availability

The datasets used and/or analyzed during the current study are available from the corresponding author on reasonable request.

## Abbreviations

EMS: Emergency Medical Services
RTIs: Road Traffic Injuries
QMIs: Quality Measurement Indicators
LMICs: Low- and Middle-Income Countries
DEMMCs: Disaster and Emergency Medical Management Centers
