# Supplementary material for: Development of quality indicators to measure pre-hospital emergency medical services for road traffic injury
Source: BMC Health Serv Res. 2021 Mar 16;21:235. doi: 10.1186/s12913-021-06238-1 (PMC7970773; doi:10.1186/s12913-021-06238-1)
Supplement: Supplementary file 1 — Additional file 1. Search strategy up to June 13st, 2020. [file 12913_2021_6238_MOESM1_ESM.docx]

**Additional file1. Search strategy**

**up to June 13^st^, 2020**

| **Search strategy in PubMed** | | |
| --- | --- | --- |
| Search #1 | ((((((((((((Emergency Medical Service[Title/Abstract]) OR (First aid[Title/Abstract])) OR (Ambulance providers[Title/Abstract])) OR (Ambulance service[Title/Abstract])) OR (Ambulance transport*[Title/Abstract])) OR (Emergency Paramedic*[Title/Abstract])) OR (First responder*[Title/Abstract])) OR (Out of hospital[Title/Abstract])) OR (Pre-Hospital[Title/Abstract])) OR (Prehospital care[Title/Abstract])) OR (Prehospital Emergency Care[Title/Abstract])) OR (Rescue worker*[Title/Abstract])) OR (Emergency Care[Title/Abstract]) | Result #  24,486 |
| Search #2 | road[Title]) OR traffic[Title]) OR injury[Title]) OR accident[Title]) OR crash[Title]) OR collision[Title]) OR Motor Vehicles[Title]) OR motorcycle[Title]) OR pedestrians[Title]) OR car[Title]) OR automobile[Title] | Result #  199,694 |
| Search #3 | (((((((indicator[Title])) OR (index[Title])) OR (assessment[Title])) OR (evaluation[Title])) OR (monitoring[Title])) OR (control[Title])) OR (survey[Title]) | Result #  988,678 |
| Search #4  article | **Search #1 AND Search #2 AND Search #3**  ((((((((((((((Emergency Medical Service[Title/Abstract]) OR (First aid[Title/Abstract])) OR (Ambulance providers[Title/Abstract])) OR (Ambulance service[Title/Abstract])) OR (Ambulance transport*[Title/Abstract])) OR (Emergency Paramedic*[Title/Abstract])) OR (First responder*[Title/Abstract])) OR (Out of hospital[Title/Abstract])) OR (Pre-Hospital[Title/Abstract])) OR (Prehospital care[Title/Abstract])) OR (Prehospital Emergency Care[Title/Abstract])) OR (Rescue worker*[Title/Abstract])) OR (Emergency Care[Title/Abstract]) AND ((fft[Filter]) AND (english[Filter]) AND (1990:2020[pdat]))) AND (road[Title]) OR traffic[Title]) OR injury[Title]) OR accident[Title]) OR crash[Title]) OR collision[Title]) OR Motor Vehicles[Title]) OR motorcycle[Title]) OR pedestrians[Title]) OR car[Title]) OR automobile[Title] AND ((fft[Filter]) AND (english[Filter]) AND (1990:2020[pdat])))) AND ((((((((indicator[Title])) OR (index[Title])) OR (assessment[Title])) OR (evaluation[Title])) OR (monitoring[Title])) OR (control[Title])) OR (survey[Title]) AND ((fft[Filter]) AND (english[Filter]))) | Result #  8,848 |

| **Search strategy in SCOPUS:**  **up to June 13^st^, 2020** | | |
| --- | --- | --- |
| Search #1 | ( ( ( ( ( ( ( ( ( ( ( ( emergency AND medical AND service[title/abstract] ) OR ( first AND aid[title/abstract] ) ) OR ( ambulance AND providers[title/abstract] ) ) OR ( ambulance AND service[title/abstract] ) ) OR ( ambulance AND transport*[title/abstract] ) ) OR ( emergency AND paramedic*[title/abstract] ) ) OR ( first AND responder*[title/abstract] ) ) OR ( out AND of AND hospital[title/abstract] ) ) OR ( pre-hospital[title/abstract] ) ) OR ( prehospital AND care[title/abstract] ) ) OR ( prehospital AND emergency AND care[title/abstract] ) ) OR ( rescue AND worker*[title/abstract] ) ) OR ( emergency AND care[title/abstract] ) | Result #  12,733 |
| Search #2 | road[title] OR traffic[title] OR injury[title] OR accident[title] OR crash[title] OR collision[title] OR motor AND vehicles[title] OR motorcycle[title] OR pedestrians[title] OR car[title] OR automobile[title] | Result #  26,417 |
| Search #3 | indicator[title] OR index[title] OR assessment[title] OR evaluation[title] OR monitoring[title] OR control[title] OR survey[title] | Result #  230,607 |
| Search #4 | **Search #1 AND Search #2 AND Search #3**  ( ( ( ( ( ( ( ( ( ( ( ( ( emergency AND medical AND service[title/abstract] ) OR ( first AND aid[title/abstract] ) ) OR ( ambulance AND providers[title/abstract] ) ) OR ( ambulance AND service[title/abstract] ) ) OR ( ambulance AND transport*[title/abstract] ) ) OR ( emergency AND paramedic*[title/abstract] ) ) OR ( first AND responder*[title/abstract] ) ) OR ( out AND of AND hospital[title/abstract] ) ) OR ( pre-hospital[title/abstract] ) ) OR ( prehospital AND care[title/abstract] ) ) OR ( prehospital AND emergency AND care[title/abstract] ) ) OR ( rescue AND worker*[title/abstract] ) ) OR ( emergency AND care[title/abstract] ) ) AND ( road[title] OR traffic[title] OR injury[title] OR accident[title] OR crash[title] OR collision[title] OR motor AND vehicles[title] OR motorcycle[title] OR pedestrians[title] OR car[title] OR automobile[title] ) AND ( indicator[title] OR index[title] OR assessment[title] OR evaluation[title] OR monitoring[title] OR control[title] OR survey[title] ) | Result #  224 |
