# Supplementary material for: Development of quality indicators to measure pre-hospital emergency medical services for road traffic injury
Source: BMC Health Serv Res. 2021 Mar 16;21:235. doi: 10.1186/s12913-021-06238-1 (PMC7970773; doi:10.1186/s12913-021-06238-1)
Supplement: Supplementary file 2 — Additional file 2. Interview Guide. [file 12913_2021_6238_MOESM2_ESM.docx]

**Interview Guide:**

1. In general, what is your opinion about evaluating the performance of pre-hospital emergency medical services for road traffic injuries?
2. What indicators do you suggest in the domain of managerial?
3. What indicators do you suggest in the domain of structural?
4. What indicators do you suggest in the domain of performance?
5. What indicators do you suggest in the other domain?
