# Supplementary material for: Development of quality indicators to measure pre-hospital emergency medical services for road traffic injury
Source: BMC Health Serv Res. 2021 Mar 16;21:235. doi: 10.1186/s12913-021-06238-1 (PMC7970773; doi:10.1186/s12913-021-06238-1)
Supplement: Supplementary file 3 — Additional file 3. List of review document. [file 12913_2021_6238_MOESM3_ESM.docx]

| **Additional file2: list of review document** | | | |
| --- | --- | --- | --- |
| **Author/s, Year: country** | **Document Type** | **title** | **Number of indicators** |
| Mehmood A, et al , 2018: USA [1] | Article | Assessment of pre-hospital emergency medical services in low-income settings using a health systems approach | 14 |
| Memongkol N, et al, 2009: Thailand [2] | Article | Development of Performance Indicators in Operational Level for Pre-hospital EMS in Thailand | 29 |
| Howard I, et al,2019: South African[3] | Article | Identifying quality indicators for prehospital emergency care services in the low to middle income setting: The South African perspective. | 14 |
| Moore L, 1999: USA[4] | Article | Measuring Quality and Effectiveness of Prehospital EMS | 18 |
| Greenberg MD, et al, 1997: USA[5] | Article | Quality indicators for out-of-hospital emergency medical services: The paramedics' perspective | 18 |
| Rosengart MR, et al, 2007: USA[6] | Article | The Identification of Criteria to Evaluate Prehospital Trauma Care Using the Delphi Technique | 28 |
| Health Information and Quality Authority (HIQA), 2012: Ireland [7] | Report | Pre-hospital emergency care key performance  indicators for emergency response times | 6 |
| Saburie E, et al, 2015:Iran [8] | Article | The Evaluation of prehospital emergency performance indicators in Birjand, 2015 | 13 |
| Iran National Emergency Organization[9] | Report | [pre-hospital evaluation indicators] | 29 |
| Christensen EF, et al,2016: Danish [10] | Article | The Danish quality database for prehospital emergency medical services | 9 |
| Kjøllesdal and Olsen, 2017[11] | Article | Developing EMS Quality Indicators in Nordic Countries | 29 |

1. Mehmood A, Rowther AA, Kobusingye O, Hyder AA: **Assessment of pre-hospital emergency medical services in low-income settings using a health systems approach**. *International journal of emergency medicine* 2018, **11**(1):53.

2. Memongkol N, Sinthavalai R, Seneeratanaprayune N, Ounsaneha W, Choosuk C: **Development of Performance Indicators in Operational Level for Pre-hospital EMS in Thailand**. *World Academy of Science, Engineering and Technology* 2009, **58**:360-365.

3. Howard I, Cameron P, Wallis L, Castrén M, Lindström V: **Identifying quality indicators for prehospital emergency care services in the low to middle income setting: The South African perspective**. *African Journal of Emergency Medicine* 2019, **9**(4):185-192.

4. Moore L: **Measuring quality and effectiveness of prehospital EMS**. *Prehosp Emerg Care* 1999, **3**(4):325-331.

5. Greenberg MD, Garrison HG, Delbridge TR, Miller WR, Mosesso VN, Roth RN, Paris PM: **Quality indicators for out-of-hospital emergency medical services: The paramedics' perspective**. *Prehospital Emergency Care* 1997, **1**(1):23-27.

6. Rosengart MR, Nathens AB, Schiff MA: **The Identification of Criteria to Evaluate Prehospital Trauma Care Using the Delphi Technique**. *Journal of Trauma and Acute Care Surgery* 2007, **62**(3).

7. **Health Information and Quality Authority. Pre-hospital Emergency Care Key Performance Indicators for Emergency Response Times: October 2012 (Version 1.1). Dublin: Health Information and Quality Authority; 2012.**

8. Saburie E, Naderi Moghadam M, Saburie O, Mohammadi Y, Tavakkoli F: **[The Evaluation of prehospital emergency performance indicators in Birjand, 2015]**. *Iranian Journal of Emergency Care* 2017, **1**(1):61-68.

9. **Performance indicators of the country's emergency organization** [<http://ems.qums.ac.ir/Portal/home/?690045/%D8%AF%D8%B3%D8%AA%D9%88%D8%B1%D8%A7%D9%84%D8%B9%D9%85%D9%84-%D9%87%D8%A7%DB%8C-%D9%88%D8%A7%D8%AD%D8%AF-%D8%A7%D8%B9%D8%AA%D8%A8%D8%A7%D8%B1-%D8%A8%D8%AE%D8%B4%DB%8C>]

10. Christensen EF, Berlac PA, Nielsen H, Christiansen CF: **The Danish quality database for prehospital emergency medical services**. *Clinical epidemiology* 2016, **8**:667.

11. Kjøllesdal JK, Olsen S: **Developing EMS Quality Indicators in Nordic Countries**. *J Emerg Med Serv* 2017, **12**(42).
